# A successful remote patient monitoring program for diabetes

**DOI:** 10.3389/fendo.2025.1524567

**Published:** 2025-02-10

**Authors:** Katlyn Sawyer, David Saxon, Richard Zane, Hemali Patel, Michael McDermott, Vatsala Singh, Helen M. Lawler

**Affiliations:** ^1^ Department of Medicine, University of Colorado School of Medicine, Aurora, CO, United States; ^2^ Division of Endocrinology, Metabolism, and Diabetes, University of Colorado School of Medicine, Aurora, CO, United States; ^3^ Endocrinology Section, Rocky Mountain Veterans Affairs Medical Center, Aurora, CO, United States; ^4^ Department of Emergency Medicine, University of Colorado Hospital School of Medicine, Aurora, CO, United States

**Keywords:** diabetes mellitus, remote patient monitoring (RPM), technology in diabetes, cloud based management, health care program, diabetes management

## Abstract

The prevalence of diabetes continues to rise in the United States along with a shortage of endocrinologists. One proposed solution to this challenge is to deliver more specialty health care services through remote patient monitoring (RPM). Here, we describe our initial experience with an RPM program for diabetes care at the University of Colorado. We enrolled 211 patients with primarily uncontrolled type 2 diabetes into the Diabetes Home and Remote Care Program (DHRCP). Remote care replaced traditional brick-and-mortar care while patients were enrolled. A certified diabetes care and education specialists (CDCES) contacted patients every 1-2 weeks to provide lifestyle coaching and assess medication compliance. With oversight from an endocrinologist, frequent medication adjustments were made by the CDCES. Analysis performed on 106 (50.2%) patients who met graduation criteria and had a hemoglobin A1c (HbA1c) completed upon program graduation showed an average decrease in HbA1c from 10.4% to 7.0% (p<0.001). Overall, our results demonstrate that RPM is an effective care model for improving glycemic control in patients with diabetes.

## Introduction

The widening gap between supply and demand for endocrine care is concerning. This gap has been attributed to a drastic rise in the prevalence of type 2 diabetes mellitus (T2D), obesity, and other endocrine conditions, overburdened primary care providers leading to inefficient utilization of endocrinology referrals, and lower compensation for endocrinologists in relation to other subspecialties contributing to fewer residents choosing to subspecialize in endocrinology (1). Over the past year, we received 21,892 referrals to our endocrinology clinic of which 40% of these referrals were for T2D. Due to high demand, there is a long wait time for new patients to be seen with only a 17% success rate in scheduling patients within 2 weeks of referral placement and at least a 3 month wait for scheduling return patients. Limitations to faster endocrine clinic access for patients include issues with space and a limited number of exam rooms as well as a finite number of endocrine providers. The Diabetes Home and Remote Care Program (DHRCP) was created as a remote care program to combat the inability of our endocrine clinic to keep up with the increased volume of referrals for T2D management and help mitigate the space constraints and endocrine provider shortage occurring in our brick-and-mortar clinic. By offloading appropriate referrals with diabetes mellitus to remote care instead of in-person care, our goal was to improve access for patients who prefer or need in-person visits while providing equivalent or better care through RPM for patients with diabetes. Recent studies have shown providers only require a median time of 10 minutes per month to successfully manage a patient with diabetes using RPM (2). This is less time than a typical clinic appointment, thus utilizing an RPM program could provide care to a higher volume of patients while also allowing providers to maximize their clinic time.

## Method

### Overview of the diabetes home and remote care program

In September 2020, to improve access and care, we launched the DHRCP for patients primarily with type 2 diabetes mellitus (T2D) with a hemoglobin A1c (HbA1c) ≥ 8.0% despite receiving regular outpatient diabetes care. Referrals to the program were accepted from endocrine providers, primary care providers (PCPs), and an inpatient glucose management team at the University of Colorado Hospital - Anschutz Medical Campus. We enrolled patients with suboptimal glycemic control despite frequently attending in-person visits for diabetes care and adhering to their prescribed medications. While in the DHRCP, patients were not seen in the brick-and-mortar clinic as this was a key component to addressing the challenges of the endocrinology provider shortage and limited exam rooms.

A certified diabetes care and education specialist (CDCES) conducted an initial phone intake to assess medications, dietary habits, and exercise. Patients were given comprehensive dietary advice based on the American Diabetes Association (ADA) recommendations with instruction provided regarding adequate fiber intake, determining glycemic index (GI), and utilizing the diabetes plate method; the Dietary Approaches to Stop Hypertension (DASH) Eating Pattern and/or the Mediterranean-Style Eating Pattern were also reviewed with patients (3). Physical activity recommendations based on the World Health Organization (WHO) guidelines were also discussed with participants where they were encouraged to partake in 150-300 min of moderate-intensity, or 75-150 min of vigorous-intensity physical activity, or some equivalent combination of moderate-intensity and vigorous-intensity aerobic physical activity, per week (4). HbA1c goals (under 7.0% or 7.5%) and/or continuous glucose monitor (CGM)/glucometer glucose goals were then set with patients. Once patients were enrolled in DHRCP, Bluetooth-enabled glucometers or cloud-based platforms Vivify Health, Codex, and Masimo were used to link patients’ glucose data from their mobile digital devices to a medical portal. If patients used CGMs, their glucose data was obtained from a cloud-based platform (i.e. – Libreview for Freestyle Libre, Clarity for Dexcom). The CDCES contacted patients every 1-2 weeks by phone to review and discuss patients’ food choices and exercise/activity in detail, reinforce lifestyle modifications, analyze blood glucose data, and adjust current diabetes medications or add pharmacotherapy under the supervision of an endocrinologist. The decision to make medication changes during each telephone interaction was made based off of glucometer or CGM data. Target fasting blood sugars were generally 80mg/dL - 130mg/dL and target post-prandial blood glucoses were < 200mg/dL. For patients with CGMs, we also set individualized time in range goals ranging between 50-70%. Diabetes technicians monitored patient glucose data in the portal for significant hyperglycemic or hypoglycemic events. These events triggered unscheduled CDCES phone calls to participants to promptly assist patients. This model allowed for more frequent touchpoints, education, and medication adjustments for patients than our traditional brick-and-mortar clinic as they received weekly care as opposed to care every 3 months. In addition, since these patients were not seen in the endocrinology clinic while in the DHRCP, this allowed in-person access for other patients.

Participants were encouraged to stay enrolled in DHRCP for as long as it took to reach their HbA1c goal and/or blood glucose goals. While enrolled in the program, primary management of diabetes was carried out by the DHRCP team. Participants were considered “graduated” from DHRCP once HbA1c and/or blood glucose levels were at their individualized goal on a stable dose of pharmacotherapy with no significant hypoglycemic episodes. At that time, participants returned to traditional face-to-face diabetes clinical care.

### Patient population

Patients were required to have a hemoglobin A1c (HbA1c) ≥ 8% to be enrolled as we targeted patients who were failing traditional care in an endocrinology or primary care clinic. Due to a lack of interpretation services, only English-speaking patients with access to a smartphone were enrolled. On average, participants were 55.2 years old, 43.1% male, 55.0% white, and 28.4% Hispanic or Latino. 92.9% of participants had type 2 diabetes mellitus (T2D), 3.8% of participants had type 1 diabetes, and 3.3% of participants had pancreatic injury induced diabetes. 30.8% of participants had private insurance compared to 22.7% with Medicare, 28.4% with Medicaid, and 18.0% unknown (**Table 1**). A total of 211 patients had participated in DHRCP at time of data collection. Participation size was limited by the number of certified diabetes care and education specialists available to interact with patients.

**Table 1:** Demographics of DHRCP patient population.

DHRCP Participant Demographics				
Characteristic	Total enrolled (n=211)	Graduates with final HbA1c (n=106)	Graduates, no final HbA1c (n=27)	Disenrolled (n=78)
**Age (years)**	55.2	57.5	56.1	51.7
**Male sex- no. (%)**	91 (43.1%)	43 (40.6%)	14 (51.9%)	34 (43.6%)
Race- no. (%)				
White or Caucasian	116 (55.0%)	68 (64.2%)	17 (63.0%)	31 (39.7%)
Black or African American	36 (17.1%)	16 (15.1%)	2 (7.4%)	18 (23.1%)
Asian	6 (2.8%)	3 (2.8%)	1(3.7%)	2 (2.6%)
Native Hawaiian or other Pacific Islander	4 (1.9%)	1(0.9%)	1(3.7%)	2 (2.6%)
American Indian/Alaska Native	3 (1.4%)	1(0.9%)	0 (0.0%)	2 (2.6%)
Other	46 (21.8%)	17 (16.0%)	6 (22.2%)	23 (29.5%)
Ethnicity- no. (%)				
Non-Hispanic, Latino/a, or Spanish Origin	149 (70.6%)	77 (72.6%)	21 (77.8%)	51 (65.4%)
Hispanic, Latino/a, or Spanish Origin	60 (28.4%)	28 (26.4%)	6 (22.2%)	26 (33.3%)
Unknown	2 (1.0%)	1 (0.9%)	0 (0.0%)	1 (1.3%)
Diagnosis- no. (%)				
Diabetes mellitus, type 2	196 (92.9%)	101 (95.3%)	23 (85.2%)	72 (92.3%)
Diabetes mellitus, type 1	8 (3.8%)	3 (2.8%)	2 (7.4%)	3 (3.8%)
Pancreatic injury induced diabetes	7 (3.3%)	2 (1.9%)	2 (7.4%)	3 (3.8%)
Days in program (#)				
Mean	147	161	243	94
Insurance- no. (%)				
Medicare	48 (22.7%)	30 (28.3%)	7 (25.9%)	11 (14.1%)
Medicaid	60 (28.4%)	24 (22.6%)	8 (29.6%)	28 (35.9%)
Private	65 (30.8%)	36 (34.0%)	8 (29.6%)	21 (26.9%)
Uknown	38 (18.0%)	16 (15.1%)	4 (14.8%)	18 (23.1%)

### Statistical analysis

A total of 211 patients with an initial hemoglobin A1C (HbA1c) ≥8.0% were enrolled in DHRCP; 133 patients (63.0%) met graduation criteria by having blood sugars at goal and/or a HbA1c at goal and a stable medication regimen; 106 patients (50.2%) who graduated from the program had a final HbA1c collected at graduation; 27 (12.8%) patients graduated from the program due to maintaining blood sugars at goal but did not have a final HbA1c collected. There were 78 patients (37.0%) disenrolled from the program either due to adherence issues or request to be removed from the program. Of the 106 patients who graduated from the DHRCP with a final HbA1c collected, 37 patients had a repeat HbA1c completed within a year of graduation.

Analysis was performed on the 106 patients who had a final HbA1c collected at graduation; a sub-analysis was completed on the 37 patients who had a repeat HbA1c within a year of graduation. A paired t-test was run on Excel to review the data and a Cohen’s d effect was used to quantify the size of the effect of this remote program. Means (standard deviations) or medians (interquartile ranges) are presented for continuous variables, and n (percents) are presented for categorical variables. Average HbA1c at enrollment, at graduation, and within one year after graduation were analyzed using two-tailed paired t-tests.

## Results

**Figure 1:**
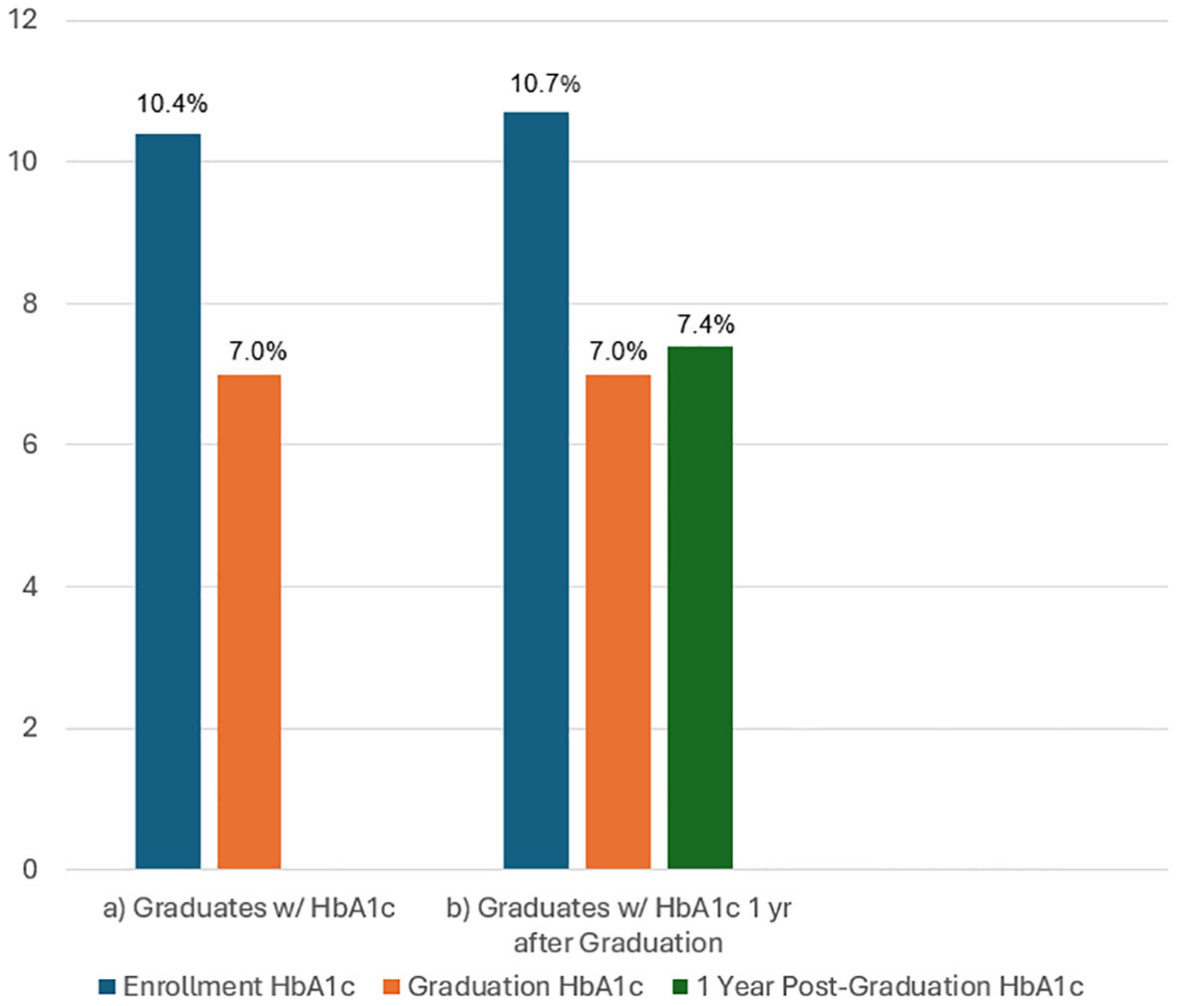
**(A)** Results of HbA1c pre-enrollment and post-graduation from the DHRCP in 106 participants and **(B)** Results of HbA1c pre-enrollment, post-graduation, and 1 year after graduation in 37 of those participants who had 1 year follow-up.

Final analysis was completed on the 106 participants who graduated from the program and had a recorded final HbA1c at graduation. Participants saw a statistically significant drop in HbA1c after completing the program with a mean initial HbA1c of 10.4% compared to a mean graduation HbA1c of 7.0% (p=<0.001; Cohen’s d of 1.529). The average length of time spent in the program to reach graduation was 161 days (median 148 days, range 49 to 371 days). A subgroup of 37 patients with HbA1c within a year of DHRCP graduation were analyzed. Their mean A1C upon entry to the program was 10.7%. There was a statistically significant increase from graduation HbA1c of 7.0% to post-graduation HbA1c of 7.4% (p=0.006) (**Figure 1**).

## Discussion

Our real-world experience demonstrates that for select individuals, RPM may possibly be superior to traditional in-person endocrinology and primary care diabetes management. In our early experience, RPM for diabetes care demonstrated the ability to achieve excellent glycemic control while improving access to specialist expertise. Through our program, 63% of patients met graduation criteria by having blood sugars at goal and/or a HbA1c at goal on a stable medication regimen. In the 50% where a graduation HbA1c was obtained, the mean HbA1c decreased from 10.4% to 7.0% (p<0.001) with a Cohen’s d of 1.529, indicating a large clinical effect. These goals were achieved in less than 6 months on average. In comparison, a study evaluating T2D management by endocrinology providers versus PCPs in traditional outpatient clinic visits from 2006 to 2017 showed 34.5% of patients seen by endocrinology providers and 29.5% of patients seen by PCPs obtained a HbA1c goal of ≤ 7.0% (6). A recent meta-analysis of 20 randomized controlled trials showed that RPM was successful in reducing HbA1c by 0.42% (p = 0.0084) in patients with T2D over a median 180-day study period (7). Other studies across multiple specialties have demonstrated an improvement in patient care with the utilization of RPM including the Cincinnati Children’s Hospital Medical Center who developed a centralized multi-specialty pediatric RPM program (8). This program served 10 different pediatric specialties and had many positive outcomes such as high patient satisfaction rates and overall improvement in patient management (8). Still, more studies relating to access to specialty care through RPM are needed.

Several digital diabetes programs including DarioHealth, Glooko, Omada, Perry Health, Teladoc (Livongo), Verily (Onduo), Vida, and Virta have recently come under fire after an independent review found them to have little to no clinical benefit in reducing HbA1c while increasing healthcare spending (9). Most of these digital programs offer behavioral modification or nutritional ketosis, but not RPM. Only Glooko includes RPM. When Glooko is paired with telephone coaching to address both medication adherence and clinical incidents, similar to our program, it has been shown to be more effective than standard care (10). We believe utilizing RPM for medication management and replacing clinic visits with RPM which allows for more frequent “touchpoints” are the missing pieces to clinical and financial success for many of these digital diabetes programs.

Interestingly, the more frequent “touchpoints” of care provided to patients in the DHRC did not appear to increase costs. Upon review of our patients with Medicare in the program, RPM costs $133 monthly (CPT 99457, 99458, 99454) where one level 4 clinic visit (CPT 99204) costs $388. There are also potential additional costs for in-person certified diabetes care and education specialist (CDCES) visits ($55 for 30 minutes) as well as the costs associated with travel to and from the clinic. In addition, by transitioning appropriate patients from physical clinics to RPM, RPM improves healthcare access for patients who should be seen in-person and allows timely and real-time care for RPM patients. As a result, RPM plays an important role in population health in managing quality metrics, increasing access to care, and improving time to care. This is particularly important in value-based care models where systems take on risk for caring for large populations of patients.

Thus, when conventional in-person care proves insufficient, RPM initiatives can help improve access to care and help patients improve glycemic control. Within the Veterans Health Administration, the Advanced Comprehensive Diabetes Care (ACDC) program coupled a nurse-driven home telehealth program with RPM technology to make both lifestyle and pharmacotherapy changes for rural veterans with clinic-refractory, uncontrolled T2D (5). The ACDC program included 230 participants over a 3-year time frame. Their study found a decrease in HbA1c from 9.56% to 8.14% (−1.43%, 95% CI: −1.64, −1.21; *P* <0.001) in 6 months and increased patient accountability for glycemic control. Another study reported RPM guided titration of insulin led to a higher number of patients reaching optimal insulin dose at 12 weeks compared to standard clinic care (11). This study was conducted on lower income patients in an urban setting, demonstrating that RPM may eliminate healthcare barriers that have traditionally made management of chronic illnesses difficult.

There were limitations to our study. One limitation was the lack of a control group. Future studies should compare the results of patients enrolled in DHRCP to patients of similar demographics receiving standard care. Ideally, this would be performed as a prospective randomized clinical controlled trial comparing RPM care to standard care in an endocrine clinic. Yet, it is reassuring that although we focused on providing RPM to patients who were failing traditional care with an HbA1C > 8% needed to enroll, 63% of the enrolled patients met graduation criteria by having blood sugars at goal and/or a HbA1C at goal and our mean HbA1c decreased from 10.4% to 7.0% in 50.2% of the enrollees. As a comparison, one study showed only 34.5% of patients seen by endocrinology in a traditional outpatient clinic setting achieved an HbA1c goal of ≤ 7.0% (6).

Also, another limitation is a comprehensive cost analysis has not yet been performed, but we plan to complete an analysis between traditional care versus DHRCP management in the future. Another limitation is 37% of participants disenrolled from the program. The main reason for disenrollment was lack of participation as this occurred in 79% of the patients who disenrolled. Lack of participation was defined as no response from participants after six weeks of weekly phone calls or messages from our CDCES. A smaller percentage, 11% of patients, disenrolled due to needing or desiring in person care due to personal preference, change of insurance, change of provider, and pregnancy. Difficulty with the technology and cost led to disenrollment in 6% and 4%, respectively. Of the disenrolled patients, 54% were female. Insurance coverage was equally represented with 1/3 of disenrolled patients having private insurance, 1/3 having Medicaid, and 1/3 having Medicare insurance.

Our study found that HbA1c increased within one year of graduation in patients who graduated from DHRCP and returned to their usual clinic care. This is likely due to the lack of oversight after graduation. Many patients commented that the frequent check-ins from the CDCES and knowledge that their blood glucose data was being monitored by the DHRCP team helped with accountability in making good food choices and taking medications as prescribed. Although the average post-graduation HbA1c was still under 8.0%, there is concern the HbA1c could continue to increase in future years post DHRCP graduation. To address this concern, we are currently designing a tier-system for DHRCP patients allowing participants to continue with different levels of monitoring ranging from weekly CDCES check-ins to monthly check-ins based on the results of RPM glucose data.

In summary, our study contributes to the growing foundation of evidence-based medicine demonstrating RPM works and should be utilized by providers, including specialists, nationwide. We believe RPM can achieve glycemic control in targeted patient populations as shown in this study while improving access to specialty care by eliminating the need for face-to-face clinic appointments.

## Data Availability

The original contributions presented in the study are included in the article/supplementary material. Further inquiries can be directed to the corresponding author.
